# Minimally invasive oblique interbody fusion for correction of iatrogenic lumbar deformity

**DOI:** 10.3171/2020.1.FocusVid.19706

**Published:** 2020-01-01

**Authors:** Christopher Wilkerson, Vance Mortimer, Andrew T. Dailey, Marcus D. Mazur

**Affiliations:** Department of Neurosurgery, Clinical Neurosciences Center, University of Utah, Salt Lake City, Utah

**Keywords:** psoas, oblique lateral interbody fusion, OLIF, minimally invasive, retroperitoneal, video

## Abstract

Spinal instability may arise as a consequence of decompressive lumbar surgery. An oblique lumbar interbody fusion combined with pedicle screw fixation can provide indirect decompression on neural elements, stabilization of mobile spondylolisthesis, and restoration of segmental lordosis. Minimally invasive techniques may facilitate a shorter hospitalization and faster recovery than a traditional open revision operation. The authors describe the use of an anterior interbody fusion via an oblique retroperitoneal approach and posterior pedicle screw fixation to treat a 67-year-old woman who developed L3–4 and L4–5 unstable spondylolisthesis after a lumbar laminectomy.

The video can be found here: https://youtu.be/KWwGMIoDrmU.

## Transcript

### 0:25 Case description

This is a 67-year-old woman with a history of a lumbar laminectomy performed 4 years ago, who developed walking intolerance, bilateral lower extremity radiculopathy, and mechanical back pain. Imaging demonstrated the presence of L3–4, L4–5 mobile spondylolisthesis with disc degeneration and foraminal stenosis. Full-length standing spine radiographs demonstrated increased anterolisthesis when weight-bearing and a loss of segmental lordosis, measuring approximately 20° from L3 to L5. She had a high-riding iliac crest that could make access to the L4–5 disc space challenging from a direct lateral transpsoas approach. The patient’s anatomy was favorable for an oblique anterior to psoas approach with an appropriate corridor between the aorta and iliac vessels and the psoas muscle. The distance between the major arteries and the psoas should be greater than 1 cm on MRI. This distance is typically greater at the upper lumbar levels, L2–3 and L3–4.

### 1:20 Advantages to the anterior oblique approach

Advantages to the anterior oblique approach are: it avoids disruption of the psoas muscle, the working corridor is far from the lumbar plexus, the surgeon can work underneath the iliac crest to access L4–5 and L5–S1, and the surgeon can access up to the L1–2 level. For levels between L1 and L5, the incidence of major vascular injury is low, so generally an access surgeon is not needed. If working at the L1–2 or L2–3 level, the renal vessels should be anticipated as the working channel is behind the renal artery and vein. The oblique approach to L5–S1 should be treated as a separate entity due to a higher risk of vascular complication.

### 1:53 Positioning for the anterior oblique interbody fusion

The patient was placed in the right lateral decubitus position with the left side up. The head and leg rest of the standard operating table was reversed to accommodate the C-arm. The iliac crest was positioned just below the table break, and the table was flexed mildly to maximize potential access to the L4–5 disc space. Gel rolls were placed under the chest and hip for support. The right hip was flexed mildly, and the left hip was kept straight to take tension off of the psoas muscle. She was secured in this position with silk tape. The table was then adjusted so that the patient was in the true lateral position with the disc spaces orthogonal to the floor in the lateral fluoroscopic view and the pedicles aligned in the AP view. The vertebra, disc spaces, and iliac crest were marked on the patient’s skin. Our incision was placed two fingerbreadths ventral to the iliac crest in a line that projected ventrally from the disc spaces. The incision was oriented obliquely along the direction of the external oblique muscle and extended from the L3–4 disc to the L4–5 disc.

### 2:46 Surgical procedure

The skin was infiltrated with local anesthetic and incised sharply. The superficial dissection down to the external oblique fascia was performed using monopolar electrocautery, but only sharp and blunt dissection was used thereafter to avoid nerve injury. The abdominal wall musculature was split using two tonsil clamps, and the transversalis fascia was opened bluntly to access the retroperitoneal space. Ilioinguinal and iliohypograstric nerves can be encountered in the abdominal wall, which should be preserved. Starting just ventral to the iliac crest, we used blunt finger dissection in the lateral to medial direction to sweep the retroperitoneal fat and abdominal contents ventrally. By starting at a lateral location, we avoid contacting the ureter, peritoneum, or abdominal contents. The psoas muscle is palpated and, anterior to this, the ventral spine.

At our institution, we use neuromonitoring to avoid nerve palsies associated with positioning as well as to prevent lumboplexus injury or nerve irritation caused by instrumentation. The disc space is then palpated using a stimulating probe to confirm that the location is away from the lumbar plexus. The stimulating probe is passed over a finger to prevent inadvertent injury to the viscera. The probe is then gently inserted into the disc space, which should be accessed at the midline or slightly ventral to midline. The stimulating dilators are then advanced in a similar fashion and then the retractor blades. The retractor is secured to the operating table using the articulating arm. The approach can be performed mostly using palpation and fluoroscopic guidance. However, a larger opening can be made for direct visualization, although this may stretch the abdominal wall and injure superficial nerves. A pin can be inserted into the vertebral body to stabilize the retractor. The pin should be placed adjacent to the endplate to avoid the segmental artery. Fixation to the L5 vertebra should be avoided to prevent injury to the iliolumbar vein.

When the retractor blades are opened, the anterior aspect of the disc space should be visualized up until the midpoint of the vertebral body. The location can be confirmed with lateral fluoroscopy, although the retractor blades can partially obscure the view. The visible structures should be the ventral portion of the psoas muscle, the disc space, and the adjacent vertebral bodies. The ALL marks the ventral most extent of the discectomy and should be avoided unless an ALL release is planned. The sympathetic plexus may pass beside the ALL. The genitofemoral nerve may be visualized coursing along the psoas. Unless working at L5–S1, generally the major arteries and veins are not visualized.

Because we are accessing the disc space from an oblique approach, all instruments should be oriented directly orthogonal to the patient to avoid entering the spinal canal or contralateral neural foramen. Often the instruments are inserted obliquely and then rotated vertically. The disc can be removed using rongeurs, rotating cutters, curettes, or box-shaped devices. Cobb elevators can be passed across the disc space to remove cartilaginous endplates and bridging osteophytes on the contralateral side. Tight disc spaces may require sequential dilators and complete disc removal to facilitate interbody graft placement. Care is taken not to violate the vertebral endplates.

A device is selected that will span the apophyseal ring, restore disc height, and increase segmental lordosis. The device should be snug enough to facilitate ligamentotaxis, which is the mechanism that enables indirect decompression of the spinal canal and neural foramina. Bone graft material is placed within the cage. Anterior placement in the intervertebral space facilitates better correction of lordosis, whereas posterior placement provides better indirect decompression.

The pin and retractor are then withdrawn carefully while looking for any areas of bleeding. The second disc space is then accessed, and the process is repeated starting with passage of the stimulating probe over a finger to the adjacent disc space. After the second cage is implanted, the retractor is carefully withdrawn. AP and lateral images show appropriate interbody graft position with adequate indirect decompression, restoration of segmental lordosis, and restoration of spinal alignment. No surgical drains are placed. The external oblique fascia is reapproximated with absorbable suture. The superficial layers are closed in the standard multilayered fashion.

### 6:45 Pedicle screw fixation

For the second stage of the operation, we placed pedicle screws via a percutaneous technique to increase the rigidity of the construct. The patient was positioned prone on a Jackson table. We used fluoroscopy to mark the midline, and additional lines were made approximately 3 cm off midline to provide guidance for screw entry points.

The reference array was placed at the posterior superior iliac spine. The PSIS was palpated, and we cut down directly onto it. The periosteum overlying the PSIS was injected with anesthetic. An awl-type device was then used to break through the cortical bone. We then advanced the percutaneous pin a few centimeters in the direction that would be used for an iliac bolt. The reference array was attached, and an intraoperative scan was performed.

A suitable trajectory was identified using image guidance. The skin was marked appropriately, then injected with anesthetic. A stab incision was made of approximately one fingerbreadth in length. We used monopolar electrocautery to dissect through the subcutaneous tissues and incise the dorsal lumbar fascia. We then used blunt finger dissection to split the paraspinal muscles, and the facet joint/transverse process was palpated.

In this case, we used an awl-tipped screw. The screw was attached to a reduction tower. Using image guidance, the screw was advanced to the intersection of the transverse process and the facet joint. A mallet was used to advance the awl tip through the cortical bone. The screw was then advanced through the pedicle and into the vertebral body. The process was repeated bilaterally from L3 to L5. Care is taken to align each screw entry point and reduction tower to facilitate passage of rods.

Rods were then sized and positioned percutaneously. Set screws are then placed through the reduction towers and finally tightened using the torque device. An intraoperative CT scan is performed, which demonstrated appropriate positioning of all pedicle screws and interbody cages. The wounds are irrigated with antibiotic solution and closed in a standard multilayered fashion. Total estimated blood loss was 250 ml. Total operative time was approximately 4 hours.

### 8:53 Conclusion

Postoperative standing radiographs demonstrate appropriate placement of all instrumentation with reduction of anterolisthesis, adequate indirect decompression of the neural foramina, and restoration of segmental lordosis with a Cobb angle of 40° between L3 and L5. Postoperative length of stay was 3 days. She was discharged to home with improved radicular symptoms, mild postoperative back pain, and no evidence of hip flexor weakness.
